# Associations Between Change in Outside Time Pre- and Post-COVID-19 Public Health Restrictions and Mental Health: Brief Research Report

**DOI:** 10.3389/fpubh.2021.619129

**Published:** 2021-01-26

**Authors:** Sydney L. Cindrich, Jeni E. Lansing, Cassandra S. Brower, Cillian P. McDowell, Matthew P. Herring, Jacob D. Meyer

**Affiliations:** ^1^Department of Kinesiology, Iowa State University, Ames, IA, United States; ^2^The Irish Longitudinal Study of Ageing, Trinity College Dublin, The University of Dublin, Dublin, Ireland; ^3^School of Medicine, Trinity College Dublin, The University of Dublin, Dublin, Ireland; ^4^Physical Activity for Health Research Cluster, Health Research Institute, University of Limerick, Limerick, Ireland; ^5^Department of Physical Education and Sport Sciences, University of Limerick, Limerick, Ireland

**Keywords:** outside time, stress, positive mental health, COVID-19, coronavirus

## Abstract

The novel coronavirus disease 2019 (COVID-19) and associated pandemic has resulted in systemic changes to much of life, affecting both physical and mental health. Time spent outside is associated with positive mental health; however, opportunities to be outside were likely affected by the COVID-19 public health restrictions that encouraged people not to leave their homes unless it was required. This study investigated the impact of acute COVID-19 public health restrictions on outside time in April 2020, and quantified the association between outside time and both stress and positive mental health, using secondary analyses of cross-sectional data from the COVID and Well-being Study. Participants (*n* = 3,291) reported demographics, health behaviors, amount of time they spent outside pre/post COVID-19 public health restrictions (categorized as increased, maintained, or decreased), current stress (Perceived Stress Scale-4), and positive mental health (Short Warwick-Edinburgh Mental Well-being Scale). Outside time was lower following COVID-19 restrictions (*p* < 0.001; Cohen's *d* = −0.19). Participants who increased or maintained outside time following COVID-19 restrictions reported lower stress (*p* < 0.001, 5.93 [5.74–6.12], Hedges' *g* = −0.18; *p* < 0.001, mean = 5.85 [5.67–6.02], Hedges' *g* = −0.21; respectively) and higher positive mental health (*p* < 0.001, 24.49 [24.20–24.77], Hedges' *g* = 0.21; *p* < 0.001, 24.78 [24.52–25.03], Hedges' *g* = 0.28) compared to those who decreased outside time. These findings indicate that there are likely to be negative stress and mental health implications if strategies are not implemented to encourage and maintain safe time outside during large-scale workplace and societal changes (e.g., during a pandemic).

## Introduction

The COVID-19 pandemic has resulted in a global disturbance in daily living patterns. In the US, COVID-19-related public health restrictions, including isolation, stay-at-home or shelter-in-place orders, and physical/social distancing requirements, were implemented by March 13th to reduce disease transmission (1). Empirical evidence supports that public health interventions such as home quarantine after infection, restricting mass gatherings, travel restrictions, and social distancing, are associated with reduced transmission rates (2–4). However, restrictions may result in adverse unintended consequences. For example, previous reports suggest that these restrictions were accompanied by changes in health-related behaviors (e.g., decreased physical activity and increased sitting time), and poor mental health (5–11).

Spending time outside is associated with lower stress and positive mental health outcomes (12, 13). Despite these reported benefits, outside time was likely affected by COVID-19 public health restrictions that encouraged people to not leave their home unless essential. Orders varied in severity, including encouragement to stay inside, allowing time outside on one's property or in the community for necessary activities outside the home (e.g., essential work, exercise), and further “non-essential” movement in the community in some states and localities [(14, 15)]. Change in outside time (i.e., the average difference of minutes per day spent outside condensed into categorical variables) may contribute to or mitigate worsening mental health, but how COVID-19-related public health restrictions influenced the amount of outside time, and how resulting change in outside time influenced stress and positive mental health [PMH; a construct encompassing hedonic and eudaimonic well-being; Tennant et al. (16)], is unknown. Findings from such a study would inform public health messages regarding outside time, as it could be useful in mitigating stress and promoting PMH when public health restrictions are necessary to prevent the spread of disease. Therefore, this study: (1) investigated differences in self-reported outside time pre- and post-COVID-19-related public health restrictions, (2) quantified differences in current stress and PMH based on change in outside time, and (3) examined the potential interaction effects of physical activity and COVID-19 related public health restriction on outside time. We hypothesized that outside time would be significantly lower following public health restrictions, and that maintained or increased outside time would be associated with lower stress and better PMH.

## Methods

### Procedures

This study investigated cross-sectional data recorded April 3rd-April 9th from the COVID-19 and Well-being (Cov-Well) Study, a population-based survey that investigated inter-relations between COVID-19 mitigation strategies, health behaviors, and mental health. Full methods were previously published (9). Briefly, participants were recruited by convenience sampling using mass emails to Iowa State University affiliated individuals (e.g., students, faculty, staff, alumni), referrals, and posts to social media platforms. Interested participants consented and enrolled in the study by clicking an electronic link and completing a 20–30-min survey. Procedures were reviewed and approved as exempt by the local Institutional Review Board (#20-144).

The questionnaire included: demographics (e.g., gender, age, race, relationship status, children in household, current employment status, and community environment), chronic health conditions, current COVID-19-related public health restrictions being followed (i.e., self-quarantine/self-isolation, under a shelter-in-place or stay-at-home order, or social/physical distancing), pre and post-restriction moderate-to-vigorous activity (MVPA) levels, and mental health questionnaires.

### Health-Related Behaviors

To assess pre/post-restriction outside time and MVPA, participants responded to two questions (pre-COVID changes and post-COVID changes) for each behavior (outside time, moderate, and vigorous activity): “How much time on an average day have you spent [outside, in moderate activity, in vigorous activity] [before *and* since] making COVID-related behavioral changes.” For Aims 2 and 3, data were coded categorically based on outside time pre-post public-health restrictions as: increased, decreased, or maintained (i.e., the same time was reported pre- and post-restrictions) outside time, respectively. Similarly, for MVPA, participants were categorized based on meeting aerobic US Physical Activity Guidelines (i.e., 150 min of moderate activity, 75 min of vigorous activity, or an equivalent combination) [(17, 18)] pre- and post-restrictions as: increased MVPA (e.g., not meeting guidelines pre but meeting guidelines post), decreased MVPA (i.e., meeting guidelines pre but not post), maintained high MVPA (i.e., meeting guidelines pre and post), or maintained low (i.e., not meeting guidelines pre or post). For cleaning, standard International Physical Activity Questionnaire (IPAQ) data cleaning rules were followed which resulted in the exclusion of anyone reporting ≥960 min/day of MVPA. For Aim 3, participants were categorized based on the highest level of COVID-19 related public health restriction that was endorsed (self-quarantine/self-isolation > under a shelter-in-place or stay-at-home order > social/physical distancing).

### Mental Health Outcomes

Based on their previously demonstrated association with outside time (12, 13), stress and PMH were selected as outcomes. Current stress was assessed using the four-item Perceived Stress Scale-[ (Cronbach's α=0.60–0.82; (19); current sample α = 0.87) and current PMH was assessed using the seven-item Short Warwick–Edinburgh Mental Well-being Scale (α = 0.83–0.87; (20); current sample α = 0.85), with higher scores indicating higher stress and better PMH, respectively.

### Statistical Analysis

The base stats package in R was used to analyze the data. For Aim 1, the normality of the data was tested with the Shapiro–Wilks test. The results indicated a significant difference (*p* < 0.0001) between outside time pre- and post-restrictions, indicating non-normally distributed data. A Wilcoxon Signed-Rank Test was used to compare outside time pre-post COVID. For Aim 2, a one-way ANCOVA assessed differences between change of outside time (i.e., decreased, maintained, or increased) on stress (Model 1) and PMH (Model 2). *Post-hoc* comparisons using the Tukey HSD procedure were used to compare the groups for each outcome. Cohen's d (21) effect sizes were used to compare change in outside time pre-post restrictions, while Hedges' g (22) was used to compare groups based on mental health outcomes. Finally, E-Value analyses were used to analyze how strong the confounders would have to be to nullify the results of the main effect. For Aim 3, the ANCOVA models in Aim 2 were used with the addition of MVPA and COVID-19-related public health restriction as interaction terms on outside time. Alpha was set at 0.05 for all analyses.

## Results

After excluding responses with incomplete data (*n* = 127), participants (*n* = 3,291; 62% female) were predominately white (94%), resided in suburban communities (56%), married (65%), and generally healthy (i.e., 63% reported never having a chronic health condition). Participants reported following a variety of COVID-19 public health guidelines, with 17% quarantined or self-isolating, 47% sheltered in place or staying at home, and 34% social distancing. Most of the participants reported a change in their work with 41% working from home when they were not before. Supplementary Table 1 presents participant characteristics and descriptive statistics for exposures, outcomes, and covariates.

### Aim 1

Overall, outside time post-restrictions was significantly lower (W = 1318802, *p* < 0.001) than outside time pre-restrictions, though the overall effect was small (*d* = −0.19).

### Aim 2

**Figure 1:**
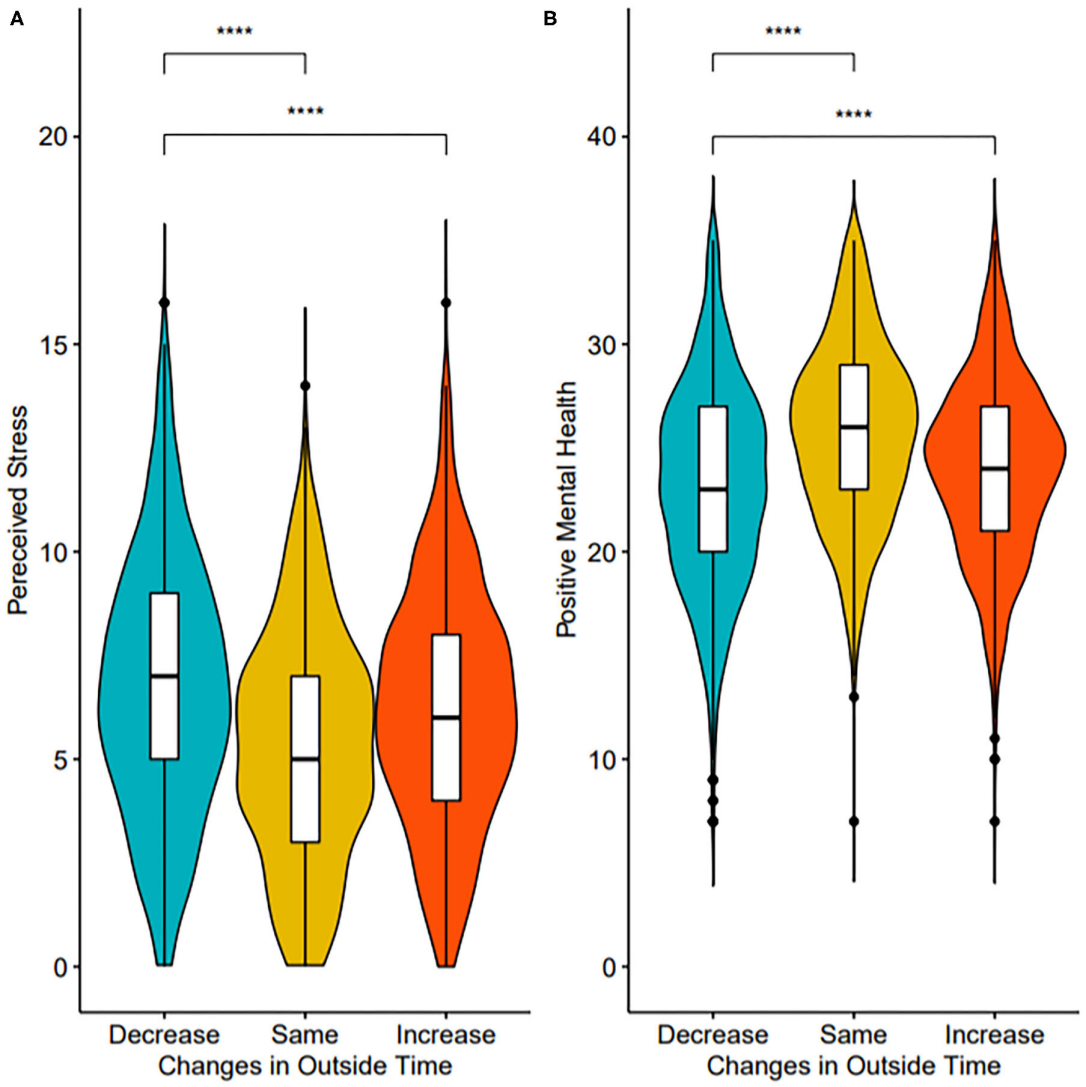
Violin and box plots of stress **(A)** and PMH **(B)** by outside time groups. Significance values indicate results of pairwise comparisons. ****p < 0.0001.

Participants were categorized into increased (*n* = 885), decreased (*n* = 1,375), or maintained (*n* = 1,031) outside time from pre to post public health restrictions. Changes in outside time were significantly associated with stress (*F*_(2,3,261)_ = 14.78, *p* < 0.001) and PMH (*F*_(2,3,261)_ = 23.78, *p* < 0.001). Compared to decreased outside time (adjusted means, stress: 6.44 [95% CI: 6.29–6.59], PMH: 23.60 [95% CI: 23.38–23.83]), Tukey's *post-hoc* tests showed increased or maintained outside time were significantly associated with lower stress (*p* < 0.001, 5.93 [5.74–6.12], *g* = −0.18; *p* < 0.001, mean=5.85 [5.67–6.02], *g* = −0.21; respectively) and significantly associated with higher PMH (*p* < 0.001, 24.49 [24.20–24.77], *g* = 0.21; *p* < 0.001, 24.78 [24.52–25.03], *g* = 0.28, respectively) (Figure 1). An E-value analysis was conducted to test how strong the unmeasured confounding variables would have to be to nullify the observed results. The results of the E-value analysis provide further support for the main effect for both stress and PMH on maintained time outside (*E*_*stress*_ = 1.56; *E*_*PMH*_ = 2.64) and increased outside time outside (*E*_*stress*_ = 1.65; *E*_*PMH*_ = 1.90). There was not a significant difference in stress or PMH for those reporting either increased or maintained outside time (*p* > 0.05). Full model results are available as supplementary material (Supplementary Table 2).

### Aim 3

The main effect of outside time remained significant with the addition of the interaction terms (MVPA and COVID-19-related public health restrictions) with outside time using Type II sum of squares for stress (*F*_(2, 3, 251)_ = 14.77, *p* < 0.001) and PMH (*F*_(2,3,251)_ = 23.76, *p* < 0.001). However, the interaction terms of outside time by MVPA and by COVID-19-related public health restrictions were not significant for stress (*F*_(6,3,251)_ = 0.64, *p* = 0.69; *F*_(4,3,251)_ = 0.86, *p* = 0.49; respectively) or PMH (*F*_(6, 3, 251)_ = 0.73, *p* = 0.63; *F*_(4,3,251)_ = 0.73, *p* = 0.57; respectively).

## Discussion

Consistent with hypotheses, our findings showed that US adults reported significantly less outside time per day following COVID-19-related public health restrictions. Moreover, maintaining or increasing outside time were associated with lower stress and higher positive mental health, regardless of the amount of physical activity engaged in or the degree of COVID-19 related public health restrictions. Transportation to and from work, social gatherings, and leisure activities often occur outside, and abrupt shifts to working from home, practicing social distancing, and limits on social gatherings likely contribute to these findings. Fear of disease transmission and attempting to comply with government mandates are additional factors that may influence the time people spend outside (23). More research is needed to understand which factors most impact outside time when public health restrictions are in place to inform public health messaging.

Moreover, individuals who were able to maintain or increase outside time each day reported less stress and higher PMH, regardless of physical activity or COVID-19 related public health restriction, although the effects were small. This is consistent with prior evidence of lower stress and improved mental health when more time is spent in a natural environment or outside (24–26). Understanding that maintaining or increasing outside time still has an effect on stress and PMH after controlling for public health restriction is especially important during the pandemic when stress may increase due to employment changes or loss, changes to childcare (e.g., online schooling), financial instability, and/or reduction of stress-mitigating hobbies (8, 27, 28) and local restrictions may need to be implemented at any time. Increasing outside time (e.g., being outdoors, walking, biking, and gardening) may be an essential component of managing stress and maintaining PMH during a global pandemic. Examining change in outside time and their association with mental health as these behaviors further change across time and in response to easing of restrictions warrants further research.

Strengths of this study are the robust sample size, psychometrically strong measures of stress and PMH, and estimates of outside time pre- and post-public health restrictions. As these were observational data, a limitation was the clarity of direction of the relationship between outside time and stress/PMH. It is plausible those high stress and poor positive mental health may lead people to be outside less. Other limitations include a predominately white and female sample, which is not an accurate representation of the total U.S. population, and retrospective report of outside time pre-COVID; therefore recall bias may have influenced these results. The state of national emergency was declared on March 13th and data was collected from April 3rd to April 9th, therefore participants had a brief recall period when self-reporting outside time prior to the restriction implementation which reduces the potential magnitude of this bias. Additionally, many participants reported a “0” value for the amount of outside time both before and after public health restrictions, which means the present effect sizes are likely to underestimate the true effect of the pandemic on outside time and its association with mental health. Finally, due to the anonymous nature of the survey, we were unable to identify possible duplicate responses or get a clear understanding of response rate and cooperation rate.

### Public Health Implications

Current findings of decreased outside time due to COVID-19 public health restrictions (coupled with previously reported reductions in physical activity and increased sedentary time), could have serious implications for the long-term mental health of the general population. The finding that increasing or maintaining outside time benefits stress and positive mental health regardless of level of physical activity or degree of COVID-19-related public health restriction in these largely active adults underlines the importance of developing strategies, programs, and messages that encourage and facilitate safe outside time throughout the current pandemic. Proposed recommendations of making parks and green spaces more accessible during the pandemic, including expanding green spaces in urban areas, installing bicycle lanes, building parks that are in closer proximity to homes, and planning for more frequent evaluations of park sanitation (29), could have a significant beneficial impact on population mental health. Implementation and evaluation of these and other strategies designed to increase outside time may be of public health value in mitigating both short-term and long-term pandemic-induced negative mental health effects.

## Data Availability Statement

The raw data supporting the conclusions of this article will be made available by the authors, without undue reservation.

## Ethics Statement

The studies involving human participants were reviewed and approved by Human-Institutional Review Board Iowa State University. The patients/participants provided their written informed consent to participate in this study.

## Author Contributions

The data collection of the project was done by CB, JM, and JL. The idea for the paper, the data cleaning, the data analysis, and the writing was done by SC and JL. The data analysis and figures were done by JM. The review of the paper and suggested ideas were done by JM, JL, MH, CM, and CB. The review and final edits of the paper were done by SC. All authors contributed to the article and approved the submitted version.

## Conflict of Interest

The authors declare that the research was conducted in the absence of any commercial or financial relationships that could be construed as a potential conflict of interest.
