# Supplementary material for: Associations Between Change in Outside Time Pre- and Post-COVID-19 Public Health Restrictions and Mental Health: Brief Research Report
Source: Front Public Health. 2021 Jan 26;9:619129. doi: 10.3389/fpubh.2021.619129 (PMC7874172; doi:10.3389/fpubh.2021.619129)
Supplement: Supplementary file 1 [file Table_1.pdf]

SUPPLEMENTARY MATERIAL

Supplementary Table 1. Characteristic and Descriptive Statistics

Demographic Variables	n (%)
Age (years)	
18-24	558 (17%)
25-34	517 (16%)
35-44	456 (14%)
45-54	405 (12%)
55-64	510 (15%)
65-74	548 (17%)
75+	297 (9%)
Gender	
Male	1216 (38%)
Female	2045 (62%)
Race	
White	3086 (94%)
Other	205 (6%)
Area of Living	
Urban	732 (22%)
Suburban	1834 (54%)
Rural	725 (22%)
Relationship	
Married/In a Relationship	2150 (65%)
Widowed	102 (3%)
Separated/Divorced	196 (6%)
Never Married	796 (23%)
Other	63 (2%)
Employment Status	
No Change in Work	834 (25%)
Working from Home	1352 (41%)
Was Not Working Before	798 (24%)
Lost employment due to pandemic	307 (9%)
Public Health Restriction	
Quarantined/Self-Isolating	591 (17%)
Shelter in Place/Stay at Home Order	1570 (47%)
Social Distancing	1130 (34%)
Physical Activity	
Maintained High	2528 (77%)
Maintained Low	238 (7%)
Increased MVPA	145 (4%)
Decreased MVPA	380 (12%)
Chronic Health Condition	
Previously	170 (5%)
Currently	1034 (31%)
Never	2087 (63%)

	Min	Median	Mean	3rd Quartile	Max
**Independent Variables (IV)**					
Outside Time[a]					
Pre-COVID	0.0	120.0	124.5	180.0	960.0
Post-COVID	0.0	60.0	103.3	120.0	960.0
**Dependent Variables (DV)**					
Stress[b]	0.0	6.0	6.1	8.0	16.0
Positive Mental Health[c]	7.0	24.0	24.2	27.0	35.0

[a]Average minutes per day before (Pre-Outside Time) and after (Post-Outside Time) implementation of COVID-19 public health restrictions.
[b]Sum of scores on the Perceived Stress Scale-4 subscales range from 1 (Never) to 5 (Very Often).
[c]Sum of scores on the Short Warwick-Edinburgh Mental Wellbeing Scale subscales range from 1 (None of the Time) to 5 (All of the Time).
