# Supplementary material for: Associations Between Change in Outside Time Pre- and Post-COVID-19 Public Health Restrictions and Mental Health: Brief Research Report
Source: Front Public Health. 2021 Jan 26;9:619129. doi: 10.3389/fpubh.2021.619129 (PMC7874172; doi:10.3389/fpubh.2021.619129)
Supplement: Supplementary file 2 [file Table_2.pdf]

Supplementary Table 2. Model 1 and 2 ANOVA results

	Model 1 (Stress)				Model 2 (PMH)			
	B	SE	t	p	B	SE	t	p
**Outside Time (ref: decreased outside time)**								
Increase Outside Time	-0.51	0.13	-4.10	<0.001*	0.89	0.19	4.67	<0.001*
Maintained Outside Time	-0.59	0.12	-5.00	<0.001*	1.17	0.18	6.56	<0.001*
**Age (ref: 18-24 yrs)**								
25-34 yrs.	-0.73	0.19	-4.00	<0.001*	0.96	0.28	3.43	<0.001*
34-44 yrs.	-0.91	0.21	-4.40	<0.001*	1.16	0.31	3.72	<0.001*
45-54 yrs.	-1.42	0.21	-6.72	<0.001*	2.11	.32	6.64	<0.001*
55-64 yrs.	-1.84	0.21	-8.90	<0.001*	3.36	0.31	10.84	<0.001*
65-74 yrs.	-2.51	0.22	-11.50	<0.001*	4.25	0.33	13.10	<0.001*
75+ yrs.	-2.32	0.26	-9.00	<0.001*	4.79	0.39	12.40	<0.001*
**Gender (ref: male)**								
Female	0.68	0.10	6.52	<0.001*	-1.07	0.15	-6.89	<0.001*
Transgender male	0.83	0.86	0.96	0.33	0.60	1.29	0.46	0.64
Transgender female	1.81	0.87	2.10	0.036*	-0.98	1.30	-0.76	0.45
Gender non-conforming	1.30	0.87	1.50	0.13	-1.55	1.30	-1.20	0.23
**Race (ref: white)**								
Non-white race	0.20	0.20	1.04	0.30	-0.06	0.29	-0.22	0.82
**Relationship Status (ref: married)**								
Widowed	-0.29	0.29	-1.02	0.31	-0.88	0.43	-2.07	0.39*
Separated/Divorced	-0.13	0.20	-0.61	0.54	-0.44	0.31	-1.43	0.15
Never Married	0.22	0.15	1.47	0.14	-0.75	0.22	-3.32	<0.001*
Other	0.76	0.36	2.12	<0.034*	-1.80	0.54	-3.35	<0.001*
Decline to Answer	0.50	0.83	0.56	0.58	0.68	1.24	0.55	0.58
**Children (ref: yes, children in household)**								
Children (no)	-0.21	0.14	-1.57	0.12	-0.26	0.20	-1.26	0.21
**COVID-19 related employment changes (ref: no change in employment)**								
Working from Home	0.20	0.13	1.60	0.11	-0.26	0.19	-1.40	0.16
Not working previously	0.12	0.15	0.82	0.41	-0.02	0.22	-0.11	0.91
Lost employment	0.99	0.19	5.26	<0.001*	-0.97	0.28	-3.43	<0.001*
**COVID-19 Public health restriction (ref: quarantine/self-isolation)**								
Shelter in Place	-0.11	0.13	-0.86	0.39	0.07	1.01	0.36	0.72
Social Distancing	-0.25	0.14	-1.75	0.08	0.12	0.21	0.60	0.55
**Activity Level (ref: maintained low)**								
Increased MVPA	-0.30	0.29	-1.05	0.29	0.61	0.43	1.41	0.16
Decreased MVPA	0.33	0.23	1.47	0.14	-1.02	0.34	-3.01	0.003*
Maintained high	-0.35	0.19	-1.86	0.06	0.40	0.28	1.45	0.15
**Community Environment (ref: urban)**								
Suburban	-0.03	0.12	-0.24	0.81	-0.11	0.18	-0.60	0.55
Rural	-0.11	0.15	-0.76	0.45	-0.01	0.22	-0.04	0.96

Ref = reference group; maintained low = not meeting MVPA guidelines pre or post COVID-19 restrictions; maintained high = meeting MVPA guidelines pre and post COVID-19 restrictions; * =p <0.05
